# Productive foraging grounds enhance maternal condition and offspring quality in a capital breeding species

**DOI:** 10.1002/ece3.70137

**Published:** 2024-09-10

**Authors:** Leila Fouda, Stuart R. B. Negus, Emma C. Lockley, Kirsten Fairweather, Artur Lopes, Anice Lopes, Sandra M. Correia, Albert Taxonera, Gail Schofield, Christophe Eizaguirre

**Affiliations:** ^1^ School of Biological and Behavioural Sciences Queen Mary University of London London UK; ^2^ University of New Brunswick Saint John New Brunswick Canada; ^3^ Associação Projeto Biodiversidade Santa Maria Cabo Verde; ^4^ Instituto do Mar, Cova de Inglesa Mindelo Cabo Verde

**Keywords:** energetic resources, foraging, loggerhead sea turtle, nesting, offspring fitness, reproduction, stable isotope analysis

## Abstract

Feeding ecology is an essential component of an organism's life, but foraging comes with risks and energetic costs. Species in which populations exhibit more than one feeding strategy, such as sea turtles, are good systems for investigating how feeding ecology impacts life‐history traits, reproduction and carried over effects across generations. Here, we investigated how the feeding ecology of loggerhead sea turtles (*Caretta caretta*) nesting at the Cabo Verde archipelago correlates with reproductive outputs and offspring quality. We determined the feeding ecology of female turtles before and during the breeding season from stable isotope analysis of carbon and nitrogen and correlated isotopic ratio with female and offspring traits. We found that female turtles feeding at higher trophic positions produced larger clutches. We also found that females with higher δ^13^C values, typical of productive foraging areas, had greater fat reserves, were less likely to be infected by leech parasites and produced heavier offspring. The offspring of infected mothers with higher δ^13^C values performed best in crawling and self‐righting trials than those of non‐infected mothers with higher δ^13^C values. This study shows adult female loggerheads that exploit productive areas build capital reserves that impact their reproductive success and multiple proxies for offspring quality. Overall, our findings provide valuable insights into the complex interplay between feeding ecology and reproductive success, and reveal the transgenerational carry‐over effects of both feeding ecology and health on offspring quality in sea turtles.

## Graphical abstract

The feeding ecology of loggerhead sea turtles plays a crucial role in shaping their reproductive outcomes and offspring quality. Through stable isotope analysis, we observed that females feeding at higher trophic positions produced larger clutches. Those with less depleted δ^13^C values, indicative of productive foraging areas, exhibited greater fat reserves, lower susceptibility to leech parasites and produced heavier offspring, suggesting that not all foraging habitats are equal and can significantly influence the fitness of sea turtle populations.

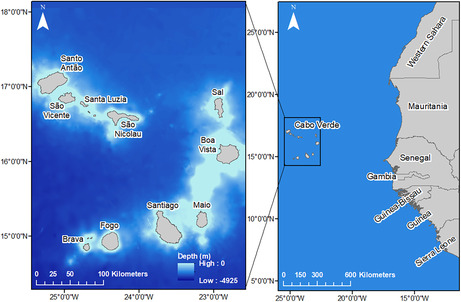

## 1 INTRODUCTION

An efficient foraging strategy is one of the most important traits for the survival and Darwinian fitness of an organism (Le Galliard et al., 2004; Stephens & Krebs, 1986). To optimise both foraging and reproductive strategies, some species have evolved long distance migrations between specific habitats (Alerstam et al., 2014; Alerstam & Bäckman, 2018). To undertake these long migrations, capital breeders require sufficient energy reserves for both migration and reproduction, unless they can complement feeding (Alerstam et al., 2014; Alerstam & Bäckman, 2018; Wheatley et al., 2008). Furthermore, capital breeding has been hypothesised to maximise energy expenditure on reproduction to produce offspring at times when they have the highest chance of recruitment (Williams et al., 2017). Therefore, if either of those hypotheses are correct, there should be a direct connection between the efficiency of foraging and reproductive success. Yet, this link often remains elusive.

In the marine environment determining feeding ecology of species is challenging (Heerah et al., 2014). Historically, direct observations and stomach content analyses, often from necropsy, have provided a snapshot of the feeding ecology of an individual, but with associated limits (Hyslop, 1980). To overcome the issues of invasive sampling, labour demand and low sample sizes, stable isotope analysis of various tissues (e.g. skin, bone) has become a method of choice, particularly for marine megafauna (Crawford et al., 2008; Pauli et al., 2017; Pethybridge et al., 2018; Post, 2002). Stable isotope analysis can be performed with minimal impacts on individuals when relying on skin biopsy or blood samples (Guiry et al., 2020; Shiffman et al., 2012) and at large scale (Newsome et al., 2009; Reich et al., 2007; Rubenstein & Hobson, 2004), expanding observations from 10s to 100s of individuals in a population. For example, stable isotope analysis has unveiled that the pre‐breeding diet quality of female Cassin's auklet (*Ptychoramphus aleuticus*) affected the timing of breeding and egg size, with individuals exploiting an energetically superior diet breeding earlier and producing larger eggs than their conspecifics (Sorensen et al., 2009).

A property leveraged by stable isotope analysis is that different body tissues have different cell turnover rates (Reich et al., 2008; Zanden et al., 2015). It is therefore possible to characterise an individual's feeding ecology across periods of its life cycle. Tissues such as teeth and bones have a slow turnover rate and therefore inform on long past feeding. The rapid turnover rate of most blood plasma cells enables the evaluation of recent feeding (within weeks). In between, skin will carry the mid‐term information of feeding, likely within months (Bearhop et al., 2002, 2004; Reich et al., 2008; Van Klinken, 1999; Vander Zanden et al., 2010). This differential turnover has been exploited to study southern elephant seals at breeding grounds (*Mirounga leonine*), with vibrissae used to identify the long‐term foraging strategies of adult females, while blood serum was used to determine fasting over the breeding and moulting periods (Hückstädt et al., 2012).

An efficient feeding ecology goes beyond an individual meeting its physiological requirements for its own survival. Parental foraging strategy, diet quality and energy reserves are directly linked to reproductive output and offspring quality and fitness (Norris et al., 2004; Sorensen et al., 2009; Warner et al., 2008). Successful reproduction is necessary for the persistence of the population and maintaining a high adaptive potential (Baltazar‐Soares et al., 2014; Clay et al., 2018; Norris, 2005; Norris et al., 2004; Sorensen et al., 2009; Warner et al., 2008). In southern elephant seals (*Mirounga leonina*), a capital breeding Phocidae, when oceanic conditions are favourable, mothers expend more energetic resources on pups, in particular male pups, than in poor environmental conditions (Fedak et al., 1996; Houston et al., 2007; McMahon et al., 2000, 2017)—directly supporting the link between diet, resource availability and reproduction.

Sea turtles are marine species with cryptic life stages that are most accessible when adult females return to their natal rookeries to nest (e.g. Cameron et al., 2019). They are considered migratory capital breeders that can cross entire ocean basins between feeding and breeding grounds, re‐migrating every two to four years depending on the time needed to reach sufficient endogenous reserves for breeding (Bonnet et al., 1998; Plot et al., 2013; Stiebens et al., 2013). Their migration and the subsequent nesting events are energetically costly from the perspectives of breeding, egg production and development, and emerging on the beach to conduct the nesting process (Marn et al., 2017). Loggerhead sea turtles (*Caretta caretta*) nest on average three to four times during the nesting season, and clutch size can vary significantly depending on female size and body condition (Miller, 1997; van Buskirk & Crowder, 1994).

Loggerhead sea turtles can exhibit dichotomous foraging strategies (Cameron et al., 2019; Hatase et al., 2010; Hawkes et al., 2006). The two main foraging strategies are defined by the habitat used as either oceanic (pelagic) or neritic (coastal). Individuals foraging in the pelagic zone mostly prey upon crustacean and gelatinous prey items in the water column (Frick et al., 2009; Hatase et al., 2010). Neritic foragers use more productive coastal environments and prey on sessile and slow‐moving benthic species such as gastropods, arthropods (Frick et al., 2009; Hopkins‐Murphy et al., 2003; Plotkin et al., 1993) and cnidaria (McClellan et al., 2010; Wallace et al., 2009).

In this study, we used loggerhead turtles nesting at the Cabo Verde archipelago to explore the impacts of foraging ecology on reproductive success. This population of sea turtles is one of the largest nesting aggregations in the world (Taxonera et al., 2022). Most turtles from this population are characterised as oceanic foragers, with some individuals exploiting areas impacted by upwelling events that enrich the baseline food web in nitrogen. A small proportion (<20%) forage along the continental shelf off Sierra Leone (Cameron et al., 2019; Hawkes et al., 2006). Unlike some other turtle aggregations, some adult females from this population can forage during the breeding period. Furthermore, during the nesting season, Cabo Verde loggerhead turtles' stable isotope values correlate with parasite infection and offspring quality (Lockley et al., 2020). It is unknown, however, whether these correlations stem from the endogenous reserves of turtles built before the breeding season, or from local supplementary feeding and associated infection risks in Cabo Verde waters.

To address these remaining knowledge gaps, we collected skin and blood samples from nesting females. We tested which of the stable isotope values of the two tissue types (skin and plasma) correlated best with a measure of capital energy (i.e. skinfold to measure body fat), their reproductive output and offspring quality. We hypothesised that if reproductive success was determined from the endogenous reserves built in the foraging ground, stable isotope values of skin tissue would correlate better with reproductive output and body condition (including infection rate) of adult nesting turtles than those of blood plasma. In contrast, if the endogenous reserves, from the foraging grounds, were less ecologically relevant than the speculated local supplementary feeding, we hypothesised that stable isotope values of plasma would correlate better with reproductive output and hatchling quality.

## 2 METHODS

### 2.1 Data collection

#### 2.1.1 Sampling nesting females

**FIGURE 1:**
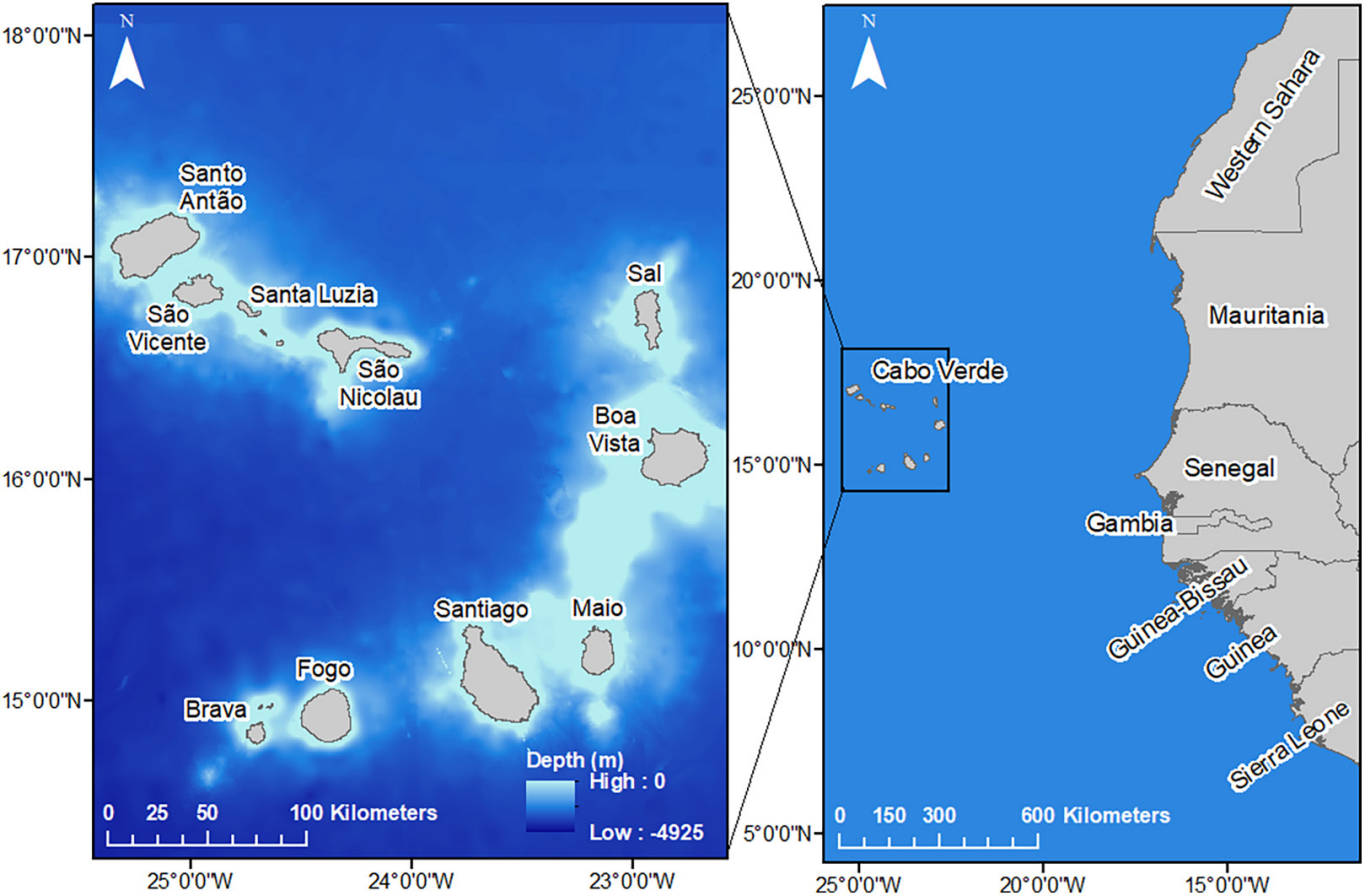
Map of the Cabo Verde archipelago located off the west coast of Africa.

Sampling took place on the island of Sal in the Cabo Verde archipelago (Figure 1) during the 2018 nesting season (July–October). To quantify long‐term change in carbon and nitrogen stable isotopes, 3 mm^2^ of non‐keratinised skin tissue was collected from the front flippers of 242 nesting females using a sterile single‐use scalpel immediately after egg deposition (Cameron et al., 2019; Stiebens et al., 2013). To investigate short‐term differences and tissue variation in carbon and nitrogen stable isotope ratios, blood samples of 1–5 mL in volume were collected from the dorsal cervical sinus of 215 females using a 40‐mm 21‐gauge needle and 5 mL syringe. Blood samples were refrigerated for up to 48 h before being centrifuged. In the field, females were tagged with a Passive Integrated Transponder (PIT) tag to enable their identification upon successive nesting events (Stiebens et al., 2013). Notch to notch curved carapace length (CCL, ± 0.1 cm) and width (CCW, ± 0.1 cm) were measured using a calibrated tape measure. Body fat was measured around the latissimus dorsi muscle using a digital calliper (accuracy ±0.1 mm) as an indication of health and endogenous reserves. Each individual was also checked for the presence of *Ozobranchus margoi*, a leech parasite known to impact reproductive output of nesting females (Lockley et al., 2020).

#### 2.1.2 Clutch sampling and offspring fitness proxy measures

To determine reproductive success, the number of eggs in each clutch was recorded and all clutches were relocated to an outdoor hatchery (Lockley et al., 2020). After emergence (50–66 days post‐oviposition), 20 hatchlings from each nest (*N*_nest_ = 88; *N*_hatchling_ = 1760) were selected at random for fitness‐related trait measurements. As hatchling size correlates with swimming performance and hatchling dispersal (Scott et al., 2014), hatchlings were measured using digital callipers from notch‐to‐notch for straight carapace length (SCL, ± 0.01 mm) and weighed (± 0.1 g), so that a Body Condition Index could be calculated:

$$\text{Hatchling Body Condition Index}=\frac{\text{Mass}\;\left(g\right)}{\mathrm{SCL}\;\left(\mathrm{mm}\right)}$$

As predator avoidance is critical for hatchling survival, two related traits were also recorded: crawl speed and self‐righting time. Crawl speed was measured by recording the time for an individual hatchling to crawl along 50 cm of flat sand, between two pieces of wood, towards a dull red light. Crawl tests were repeated twice and an average was taken (Lockley et al., 2020; Maulany et al., 2012). Self‐righting capacity was evaluated by placing a hatchling on its back on a section of flat sand and recording the time taken to right itself (Maulany et al., 2012). Self‐righting trials were repeated three times per individual, and if the hatchling took longer than 60 sec to self‐right, it was considered to have failed the trial. We measured the number of successful trials (0–3) and the average self‐righting time from successful trials in seconds.

### 2.2 Stable isotope analysis

Skin samples were washed in distilled water and dried at 60°C for 48 h. Between 0.7 μg and 1.3 μg of the sample were placed in tin capsules (4 mm) before being combusted using a continuous flow isotope ratio mass spectrometer (Integra2, Sercon, Crewe, UK), which simultaneously analysed nitrogen and carbon elements. To verify the accuracy of the readings, an internal standard of casein was run at set intervals (*n* = 10). Plasma samples were spun before being freeze‐dried for 24 h. Between 1.005 μg and 1.156 μg of the dried sample was used. For both tissue samples, the C:N ratio was relatively low (skin: 3.15 ± 0.23; plasma: 4.62 ± 0.38) indicating low bias stemming from lipid enrichment (Post et al., 2007). In addition, there was a negative correlation between C:N ratios and δ^13^C values for skin and plasma, indicating a minor effect of lipids. Carbon and nitrogen weights have been deposited with on Dryad for cross‐species comparisons (Post et al., 2007). δ^13^C and δ^15^N were calculated as followed:

$$\mathrm{\delta Χ}=\left(\left(\frac{R_{\text{sample}}}{R_{\text{standard}}}\right)-1\right)\times 1000$$

where *Χ* is ^13^C or ^15^N, *R*_sample_ gives the ratio of heavy‐light isotopes ^13^C/^12^C or ^15^N/^14^N, and *R*_standard_ gives the ratio of heavy‐light isotopes of the standard. Note, the internal (casein) standard had an analytical precision of ±0.1‰ for both δ^15^N and δ^13^C.

### 2.3 Statistical analyses

To investigate how stable isotope values changed over the nesting period and tissue type, we performed independent linear models for δ^15^N and δ^13^C values of skin and plasma. Note that increased δ^15^N is usually considered a starvation signal, while patterns are more variable and context dependent for δ^13^C (Doi et al., 2017).

Maternal health and size are known to correlate with stable isotope values in loggerhead turtles (Eder et al., 2012; Hatase et al., 2010; Lockley et al., 2020). Therefore, we ran separate linear models for both tissue types to determine the relationship between stable isotope values, maternal body fat reserves, CCL and infection status (i.e. parasite presence). Because of changes in abiotic factors (like temperature) across the nesting season, we also created a variable called ‘season period’ to split the nesting season into three periods of equal duration: early (09/07/2018 to 12/08/2018), middle (between 13/08/2018 and 16/09/2018) and late (17/09/2018 to 19/10/2018). This variable was included as an explanatory variable in these models as well as all two‐way interactions. In the data exploration phase, we found that body fat measurements correlated with CCL of the nesting turtles. Hence, residuals were used to create a measure of the adult body fat reserves that accounts for allometric scaling. Linear models were used to determine the relationship between maternal size and body fat reserves. Julian day was included in the model to examine if these relationships changed as the season progressed.

Following our working hypotheses, we tested whether stable isotope values and body fat reserves were correlated with individual clutch size using linear models and conducted separate models for skin and plasma samples. Skin sample models were used to test for the long‐term effect associated with forging area (i.e. during the non‐breeding period), and plasma sample models were used to test for the short‐term effects of possible supplementary feeding at the nesting ground. The linear models included maternal body fat reserves, CCL, parasite presence and nesting season period (early, middle, late) and all two‐way interactions as explanatory variables. Three‐way interactions with season period were also included to investigate the fixed predictor interactions as the season progresses.

We used a series of linear mixed effect models (LMMs) to test whether components of maternal feeding ecology and body fat reserves were correlated with offspring fitness proxy measurements. In all LMMs, female ID was used as a random effect to account for hatchlings relatedness. As the incubation duration can affect hatchling phenotype, it was included as a fixed predictor (Ferguson & Deeming, 1991; Hatase et al., 2018). CCL is known to correlate with clutch size and maternal investment, and therefore was also included as a fixed predictor. Separate models were run for skin and plasma tissues. Models focusing on hatchling length and mass included maternal δ^15^N and δ^13^C values, body fat reserves, CCL, parasite presence, season period and incubation duration as explanatory variables. The hatchling mass model also included SCL as an explanatory variable because larger turtles are expected to be heavier. All two‐way interactions were used as explanatory variables, as well as the three‐way interactions between stable isotope values and incubation duration. This is because of the known effect of incubation duration on phenotypic characteristics. The LMM models for crawl speed and self‐righting additionally included hatchling body condition index (Mass/SCL) to account for hatchling weight on performance.

The statistical analyses were conducted in R version 4.0.0 (R Core Team, 2020). All models were backwards selected using Akaike's information criterion (AIC). In models where fixed variables showed collinearity, we used the residuals of the regression between the two variables to replace one so that both variables remained independent in the models.

## 3 RESULTS

Blood and skin samples were collected from 215 and 242 adult females, respectively. Turtle body fat reserves were measured for 227 adult females. Among those, parasites were present on 77 (33.9%) turtles.

### 3.1 Temporal variation in stable isotope values over the nesting season

**FIGURE 2:**
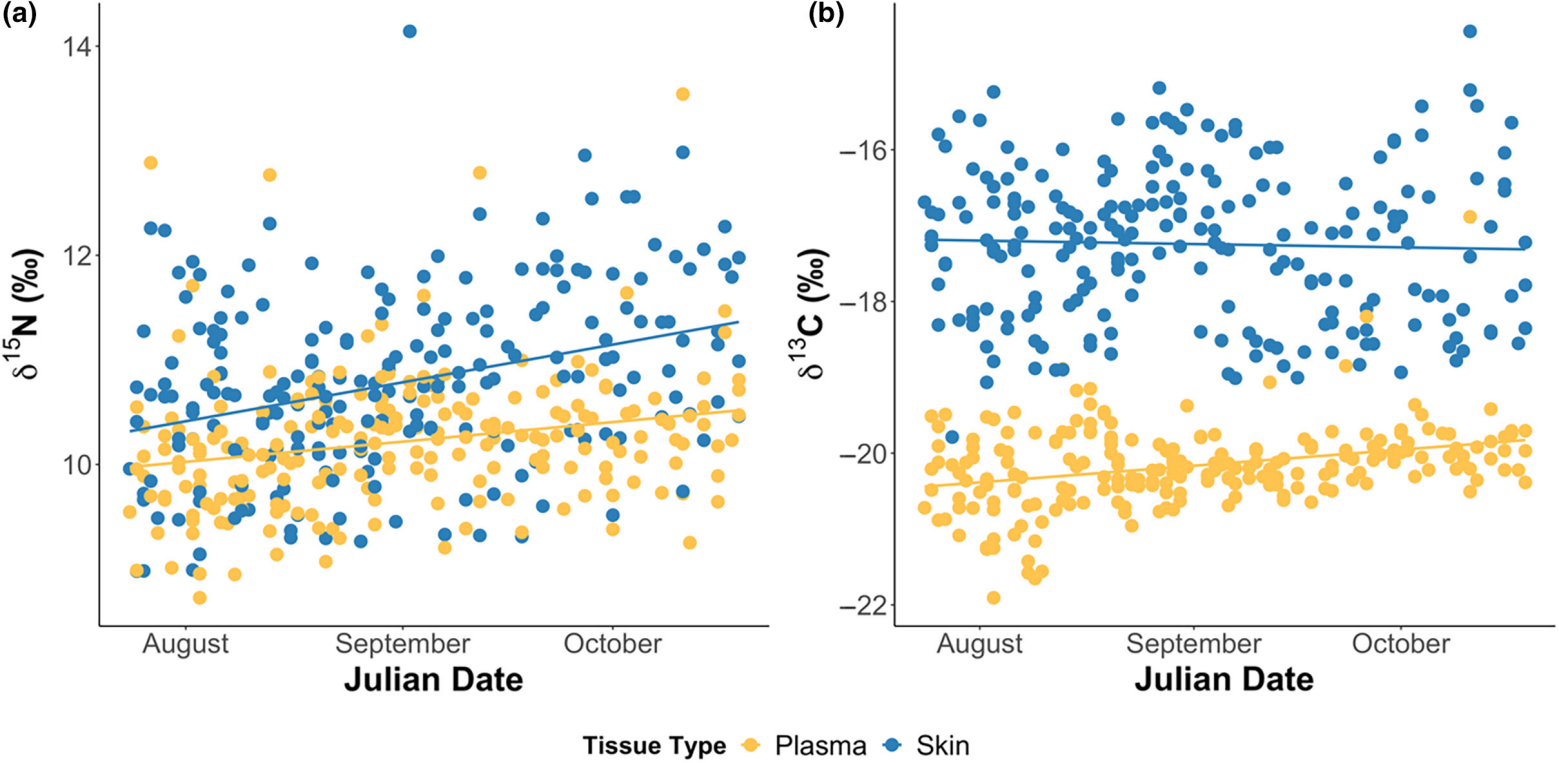
Change in stable isotope values over the 2018 nesting season. (a) Both skin (*n* = 242) δ^15^N (*F*_1,237_ = 24.675, *p* < .001) and plasma (*n* = 215) δ^15^N (*F*_1,210_ = 10.315, *p* = .002) increased over the season. (b) No trend in skin δ^13^C was seen over time (*F*_1,237_ = 0.093, *p* = .76), whereas plasma δ^13^C increased (*F*_1,210_ = 22.206, *p* < .001).

When investigating the link between temporal variation and stable isotope values, we found an interaction between tissue type and Julian day for both carbon and nitrogen isotopes (Table 1; Figure S1). δ^15^N was higher in skin tissue samples than plasma (0.6‰ SE ± 0.1 higher in skin, *F*_1,449_ = 66.579, *p* < .001), with values in both skin (*F*_1,237_ = 24.675, *p* < .001) and plasma (*F*_1,210_ = 10.315, *p* = .002) increasing over the nesting season (skin increase: 1.1‰ SE ± 0.2; plasma increase: 0.6‰ SE ± 0.2). Similarly, skin δ^13^C values were also higher than plasma δ^13^C values (2.9‰ SE ± 0.1 higher in skin, *F*_1,449_ = 1425.800, *p* < .001), but there was no significant change in skin δ^13^C in over the nesting season (*F*_1,237_ = 0.093, *p* = .76). This was unlike plasma δ^13^C values that increased over time (plasma increase: 0.7‰ SE ± 0.1; *F*_1,210_ = 22.206, *p* < .001; Figure 2, Table S1). When examining how the within‐individual differences between skin and plasma changed over the season, we found that skin δ^15^N became increasingly different to blood plasma δ^15^N (0.3‰ (SE ± 0.1) to a 0.9‰ (SE ± 0.1), *F*_1,210_ = 9.193, *p* = .003), while skin δ^13^C values became increasingly similar to blood plasma δ^13^C values (3.3‰ (SE ± 0.1) to a 2.5‰ SE ± 0.2 difference, *F*_1,210_ = 9.233, *p* = .003).

**TABLE 1:** Summary statistics for δ^15^N and δ^15^C (±SD) values in skin and plasma across the nesting season. The table includes the average stable isotope values, the C:N ratio in skin and plasma and the difference between skin and plasma values for individuals across the season.

Part of season	Average skin δ^15^N (‰)	Average plasma δ^15^N (‰)	Average skin δ^13^C (‰)	Average plasma δ^13^C (‰)	Skin δ^13^C:δ^15^N (μ)	Plasma δ^13^C:δ^15^N (μ)	Skin‐plasma δ^15^N difference (‰)	Skin‐plasma δ^13^C difference (‰)
All	10.8 (±0.5)	10.2 (±0.6)	−17.3 (±1.0)	−20.2 (±0.5)	3.2 (±0.2)	4.6 (±0.4)	0.6 (±0.8)	2.9 (±1.1)
Early	10.6 (±0.9)	9.9 (±0.7)	−17.4 (±1.0)	−20.5 (±0.6)	3.1 (±0.2)	4.7 (±0.5)	0.6 (±0.8)	3.2 (±1.1)
Middle	10.6 (±0.8)	10.3 (±0.6)	−17.2 (±1.0)	−20.1 (±0.4)	3.1 (±0.2)	4.7 (±0.4)	0.4 (±0.7)	3.0 (±1.0)
Late	11.2 (±0.8)	10.3 (±0.5)	−17.5 (±1.0)	−20.0 (±0.4)	3.2 (±0.3)	4.5 (±0.3)	0.9 (±0.79)	2.5 (±1.0)

### 3.2 Maternal characteristics

**FIGURE 3:**
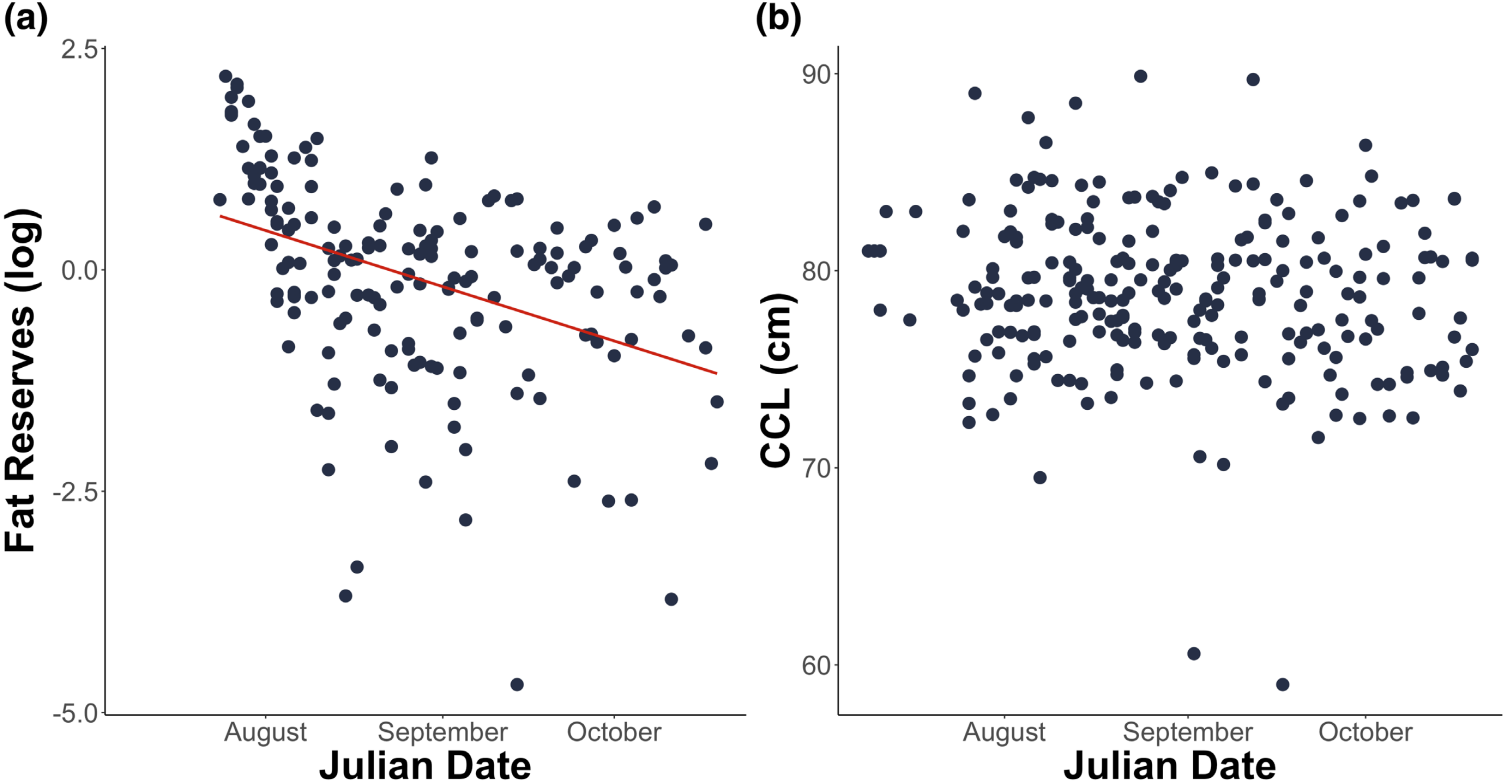
(a) Body fat reserves—the residuals of the relationship between body fat measurements against the CCL—(*n* = 227) and (b) curved carapace length (CCL; *n* = 242) of adult female loggerhead sea turtle during the 2018 nesting season on Sal Island, Cabo Verde. Body fat reserves of sea turtles significantly declined over the nesting season (*F*_1,162_ = 32.471, *p* < .001) whereas CCL showed no significant change (*F*_1,237_ = 2.818, *p* = .095), suggesting no cohort effects in the arrival to the nesting ground.

Body fat reserve measured from the fat surrounding the latissimus dorsi muscle of adult females was used as a proxy to evaluate the breeding costs of individual sea turtles. As expected from costly reproductive behaviours, we found that body fat reserves significantly decreased over the nesting season (*F*_1,158_ = 22.781, *p* < .001). The results remained identical after the removal of outliers and showed a significant decrease of 8.49 mm (SE ± 7.14) from the start to the end of the season (*F*_1,162_ = 32.471, *p* < .001; Figure 3a). In contrast, and also as expected, the CCL of adult females did not change over the season (*F*_1,237_ = 2.818, *p* = .095), suggesting that the decline in body fat reserves was not due to a concurrent arrival of a cohort of smaller turtles (Figure 3b).

### 3.3 Determinants of stable isotope variation in skin and plasma of nesting turtles

Once we established how body fat reserves varied over the course of the nesting season, we investigated the determinants of isotope variation. Skin δ^13^C values positively correlated with female CCL (0.91‰ (SE ± 0.5) difference; *F*_1,207_ = 6.665, *p* = .011; Table S2), confirming previously known patterns of larger females feeding at nutrient enriched oceanic foraging grounds (Cameron et al., 2019).

**FIGURE 4:**
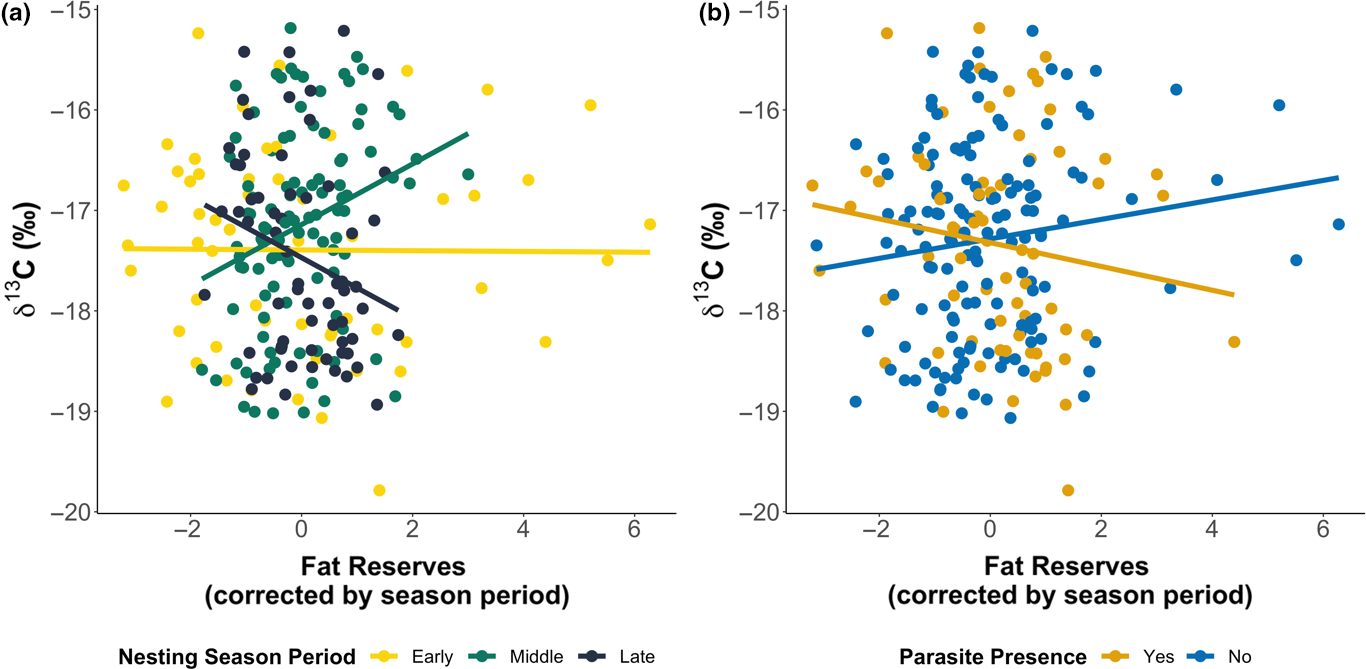
Relationship of turtle (*n* = 227) skin δ^13^C to body fat (fat reserves regressed against season period) in combination with (a) nesting season period (*F*_2,207_ = 7.695, *p* < .001) and (b) turtle infection status (*F*_1,207_ = 5.013, *p* = .026).

While at the start of the season, no clear correlation between δ^13^C and body fat reserves were found, this changed as the nesting season progressed, with a positive correlation found in the middle of the season and a negative one at the end as such turtles with greater fat reserves in the middle of the nesting season had 4.5‰ (SE ± 1.1) higher δ^13^C values (*F*_2,207_ = 7.695, *p* < .001; Figure 4a).

Investigating further the effects of turtle physiology, we found that uninfected turtles with high body fat reserves had significantly higher δ^13^C values than infected turtles (1.8‰ (SE ± 0.7) higher; *F*_1,207_ = 5.013, *p* = .026; Figure 4b), independent of the period within the nesting season. This result suggests that oceanic turtles, not linked to upwellings, are more likely to be infected by this leech parasite. Although interestingly an interaction between CCL and parasite infection on δ^15^N (*F*_1,208_ = 4.054, *p* = .045) showed larger infected turtles having higher skin δ^15^N values compared to smaller individuals (1.3‰ (SE ± 0.8) higher), suggesting large turtles feed in nitrogen‐enriched environments, but are more likely to be exposed to parasites.

When investigating the shorter term signal of turtle feeding ecology, we first found that plasma δ^13^C values increased over the course of the nesting season (0.5‰ (SE ± 0.1) increase; *F*_2,197_ = 17.715, *p* < .001). Plasma δ^15^N values, also increased as the season progressed (0.3‰ (SE ± 0.1) increase; *F*_2,196_ = 6.538, *p* = .002) and correlated with an interaction between body fat reserves and CCL (*F*_1,196_ = 16.125, *p* < .001). Over the season, plasma δ^15^N values increased and larger turtles with greater body fat reserves showed higher δ^15^N values than smaller turtles (3.4‰ (SE ± 0.8) higher than shorter turtles; 3.1‰ (SE ± 0.7) higher than lower fat turtles). Overall, variation in skin stable isotopes was explained by a combination of seasonal variation and maternal condition, including body fat reserves and infection status. Differences in plasma isotope ratios were, however, mostly related to seasonal variation.

### 3.4 Correlation of body fat reserves and foraging on reproductive output

**FIGURE 5:**
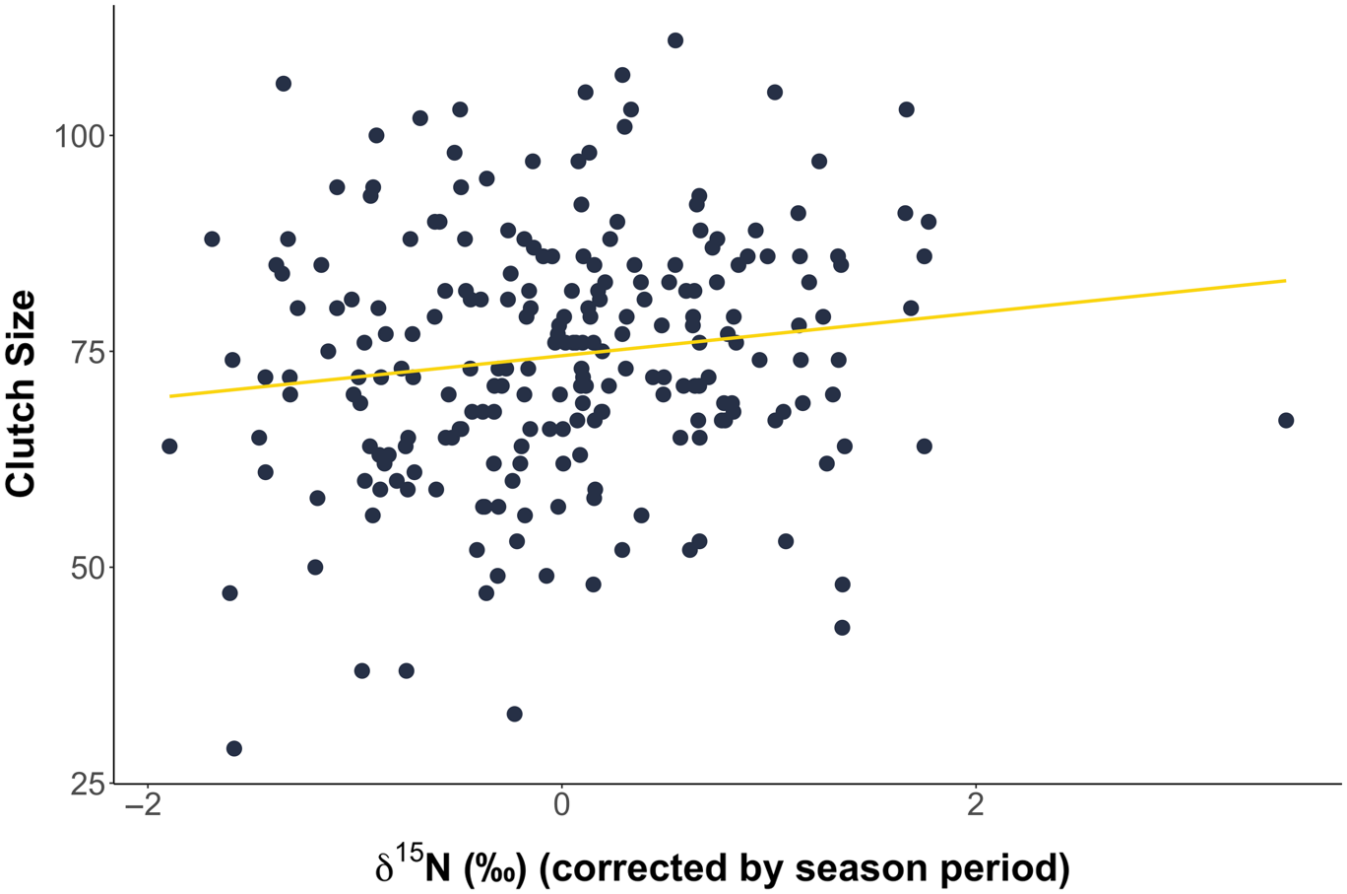
Clutch size (*n* = 226) positively correlated with δ15N values in the skin (*F*_1,199_ = 6.010, *p* = .015).

As we found a link between stable isotope values, feeding ecology and maternal conditions, we investigated the link with reproductive output. Clutch sizes ranged from 29 to 111 eggs (mean = 75, SD ± 14). As known from sea turtle research, we found that larger turtles laid more eggs (33 (SE ± 7) more) than their smaller counterparts (*N* = 226, *F*_1,224_ = 22.489, *p* < .001). Clutch size significantly decreased across the nesting season (21 (SE ± 4) egg decrease; *F*_1,224_ = 31.963, *p* < .001). Interestingly, turtles with higher skin δ^13^C values had significantly larger clutches during the early season period (30 (SE ± 8) eggs larger; *F*_2,195_ = 7.536, *p* < .001), but no differences were found later in the season. Clutch size also positively correlated with skin δ^15^N values, indicating that turtles foraging at higher trophic levels produced larger clutches (14 (SE ± 6) eggs larger; *F*_1,199_ = 6.010, *p* = .015; Figure 5). Clutch size was not correlated with either plasma δ^15^N or δ^13^C values (δ^15^N: *F*_1,187_ = 0.662, *p* = .417; δ^13^C: *F*_1,187_ = 0.029, *p* = .866), suggesting the determinant of reproductive output scales with feeding in the foraging habitat and not with possible complementary feeding.

### 3.5 Maternal condition correlates with offspring fitness proxies

By relocating clutches into an in situ hatchery, we could evaluate the link between maternal feeding ecology, reproductive output and offspring quality upon emergence. The incubation duration of nests ranged from 50 to 66 days (mean = 54.49, SD ± 3.54). Hatchlings from 88 nests representing 1760 individuals were assessed.

#### 3.5.1 Hatchling size

The length of hatchlings (SCL) was negatively correlated with maternal body fat reserves (*F*_1,73_ = 7.287, *p* = .009; Table S3) and clutch size (*F*_1,73_ = 9.972, *p* = .002) but positively correlated with adult CCL (*F*_1,73_ = 12.912, *p* < .001). Hatchlings were 2.00 mm (SE ±0.73) larger in clutches from mothers with lower fat reserves while hatchlings from smaller clutches were 2.68 mm (SE ±0.85) larger. Furthermore, we found that maternal δ^15^N and δ^13^C values of skin did not correlated with SCL (δ^15^N: *F*_1,73_ = 3.611, *p* = .061; δ^13^C: *F*_1,73_ = 1.438, *p* = .234). Maternal plasma δ^13^C values, however, were positively correlated with hatchling SCL (*F*_1,68_ = 6.979, *p* = .010), unlike plasma δ^15^N values (*F*_1,68_ = 0.417, *p* = .521). Considering the non‐significant but model‐maintained interaction between plasma δ^13^C and plasma δ^15^N offspring from mothers with higher values of plasma δ^13^C and δ^15^N was 1.69 mm (SE ±1.42) longer than mothers with more depleted plasma stable isotope values.

We found that turtle that foraged in productive oceanic grounds areas, that is, in higher skin δ^13^C values, produced heavier hatchlings (1.3 g (SE ±0.5) heavier; *F*_1,70_ = 7.149, *p* = .009) likewise in adult females with lower body fat reserves (*F*_1,1586_ = 42.526, *p* < .001). While the shortest offspring from mothers with lower body fat weighed 1.9 g (SE ±0.9) less than offspring from mothers with the highest body fat, the longest offspring of mothers with lower body fat were 5.0 g (SE ±0.8) bigger than offspring from mothers with the highest body fat. Hatchling mass was not significantly correlated with skin δ^15^N values (*F*_1,70_ = 0.120, *p* = .730). When deposited in bigger clutches the offspring of larger (CCL) mothers were heavier (5.6 g (SE ±1.7)) than offspring of smaller mothers. However, when smaller clutches were deposited, the offspring of larger (CCL) mothers were lighter (2.3 g (SE ±1.9)) than those of smaller mothers (*F*_1,70_ = 5.413, *p* = .023). Indicating that, when larger turtles with the capacity to lay big clutches deposit small ones, they may have fewer resources to invest in offspring. The offspring of females with higher plasma δ^13^C were heavier—longer hatchlings were 3.3 g (SE ±0.4) heavier and shorter hatchlings were 0.7 g (SE ±1.0) heavier than offspring from mothers with lower plasma δ^13^C (*F*_1,1524_ = 11.804, *p* < .001). Whereas the offspring of females with higher plasma δ^15^N values were lighter, this was hatchling size (SCL) mediated—longer hatchlings were 3.4 g (SE ±0.9) heavier, but shorter hatchlings were 1.8 g (SE ±1.1) lighter than offspring for mothers with higher plasma δ^15^N (*F*_1,1523_ = 14.191, *p* < .001). However, this relationship was also dependent on offspring SCL. These results suggest that maternal resources, when available, are directed into hatchling size and that feeding location in the foraging grounds is important to hatchling size. However, the plasma correlations indicate that foraging habitat alone does not drive hatchling size.

#### 3.5.2 Hatchling dispersal performance

**FIGURE 6:**
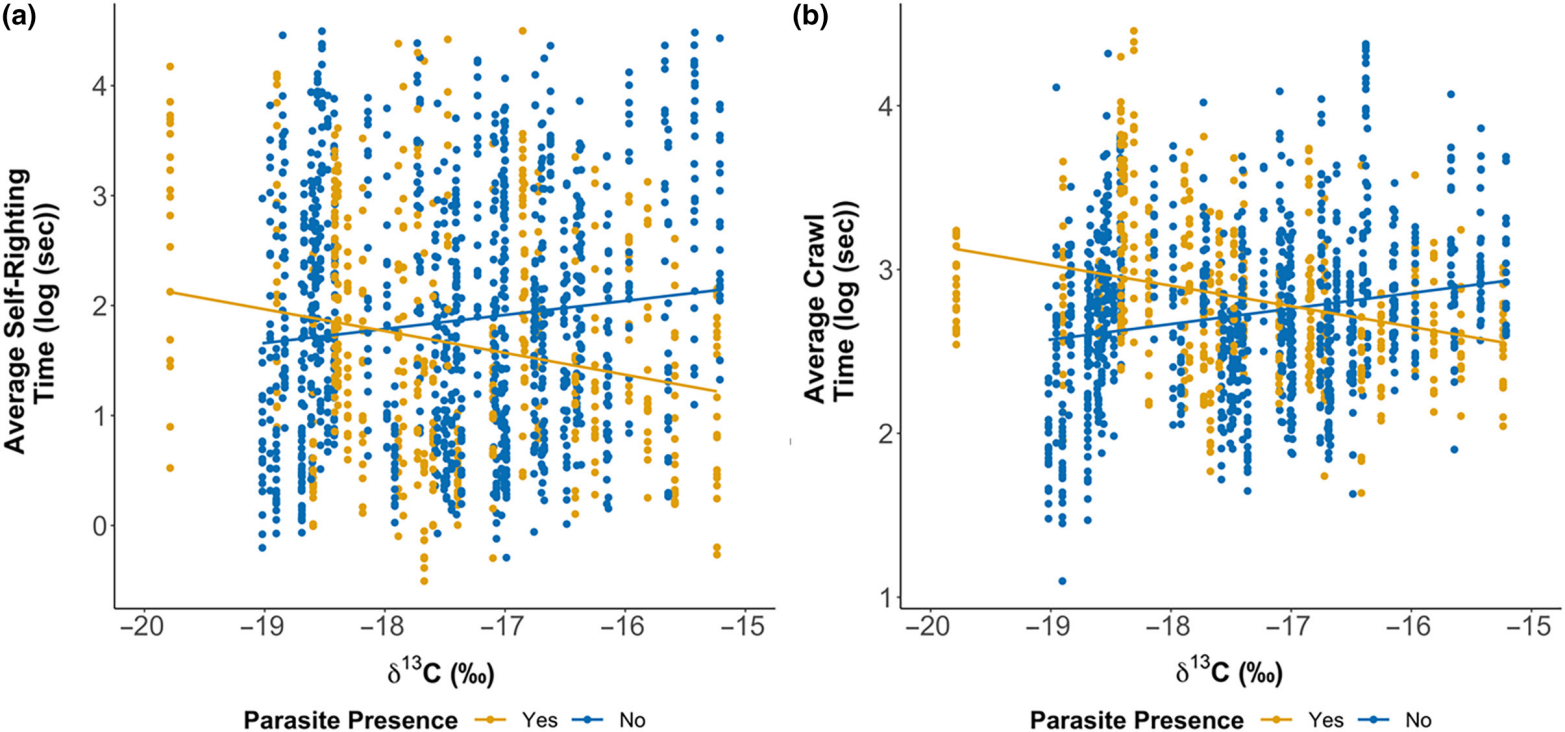
Scatterplots that show hatchling self‐righting and crawl time result with skin δ^13^C and maternal parasites presence. We found that offspring of infected mothers were faster in (a) self‐righting (*F*_1,69_ = 5.622, *p* = .021) and (b) crawl speed (*F*_1,69_ = 7.814, *p* = .007) tests.

Hatchling dispersal performance in fitness tests was mostly linked to maternal skin δ^13^C. Hatchling crawl speed and self‐righting times were negatively correlated with hatchling body condition index (crawl speed: *F*_1,1267_ = 5.655, *p* = .018; self‐righting: *F*_1,1190_ = 6.003, *p* = .014). Smaller hatchlings had 1.5 s (SE ±0.2) and 2.6 s (SE ±1.1) slower crawl and self‐righting times respectfully. We found that offspring of infected mothers with higher skin δ^13^C were faster in crawl tests (*F*_1,69_ = 7.814, *p* = .007) and self‐righting trials (*F*_1,69_ = 5.622, *p* = .021; Figure 6). Crawl times were 1.3 s (SE ±0.2) faster and self‐righting times were 3.3 s (SE ±1.4) faster than the offspring of mother's with higher skin δ^13^C and no parasites. This suggests hatchling ability to disperse and avoid predation is linked to maternal condition and foraging habitat.

Hatchling crawl speeds were correlated with an interaction between nest incubation duration and maternal skin δ^13^C (*F*_1,69_ = 5.734, *p* = .019). Specifically, longer incubation durations for hatchlings from females with lower skin δ^13^C had slower crawl speeds that led to 2.6 s (SE ±1.0) faster crawl times. Nest incubation durations were significantly negatively correlated with self‐righting times with longer incubations leading to a 2.4 s (SE ±0.91) faster performance (*F*_1,69_ = 6.532, *p* = .013). Additionally, an interaction between female infection status and clutch size was significantly correlated with self‐righting times (*F*_1,69_ = 6.747, *p* = .011). While smaller clutches from infected females produced hatchlings with 5.2 s (SE ±2.9) faster self‐righting speeds than uninfected females, in larger clutches, the opposite was true, and hatchlings were 1.4 s (SE ±0.7) slower. Furthermore, females with lower δ^13^C plasma values had offspring with faster self‐righting times (0.3 s (SE ±0.1); *F*_1,68_ = 6.424, *p* = .014). Higher maternal plasma δ^15^N values were negatively correlated with self‐righting times resulting in 3.8 s (SE ±1.9) faster performances (*F*_1,68_ = 7.224, *p* = .009). No significant relationship was found between hatchling crawl times and plasma δ^13^C or δ^15^N (δ^13^C: *F*_1,66_ = 3.655, *p* = .060; δ^15^N: *F*_1,65_ = 0.184, *p* = .669). Overall, our results show that offspring quality, examined via fitness proxies, is complex but correlates with maternal stable isotope values, from both maternal blood plasma and skin suggesting that maternal supplementary feeding and foraging ground both impact hatchling quality.

## 4 DISCUSSION

An efficient feeding ecology determines the reproductive output of individuals, and it has been hypothesised to be particularly true in migratory capital breeders where foraging and breeding grounds can be far apart. In this study, we used stable isotope analysis to test how feeding ecology and associated energy stores, in the form of body fat reserves, of adult female loggerhead sea turtles correlates with their reproductive output and the quality of their offspring. We found that δ^13^C and δ^15^N ratios obtained from skin samples, but not from plasma, were the best predictors of nesting females' reproductive output. Body fat reserves decreased as the nesting season progressed, indicating a slow depletion in endogenous reserves. Furthermore, we found that the healthiest females, those uninfected with parasites and with high body fat, had high skin δ^13^C values. Turtles with higher δ^15^N values, likely foraging in productive oceanic upwelling areas, had larger clutch sizes. Together skin δ^15^N and δ^13^C values suggest that not all foraging habitats are equal, and this difference translates into reproductive output with an impact on offspring quality. Indeed, we found that females with higher skin δ^13^C values had heavier and faster offspring. Overall, our findings not only provide valuable insights into the complex interplay between feeding ecology and reproductive success but also reveal the transgenerational carry‐over effects of both feeding ecology and health on offspring quality in sea turtles.

From skin stable isotope analysis, we found that turtles occupy mostly two different niches, linked with pelagic feeding, with one impacted by ^15^N‐enriched upwelling events (Cameron et al., 2019). This matches previous studies conducted in Cabo Verde, which identified one more feeding strategy linked to neritic habitat, but only found in turtles nesting on the island of Boa Vista. Those neritic and ocean feeding behaviours have also been confirmed by satellite tracking (Hawkes et al., 2006).

For migratory capital breeding species, resource acquisition (e.g. foraging) and resources use (e.g. migration or reproduction) are geographically segregated (Hays, 2000; Kwan, 1994; Perrault et al., 2014). As expected, the body fat reserves of female nesting turtles decreased as the nesting season progressed. Previous studies estimated that turtles weigh roughly 25% more at the foraging grounds than at the breeding grounds (Hays & Scott, 2013; James et al., 2005) and we show continuous reserve loss over the nesting season. Our result is consistent with repeated measurements of nesting female green turtles (*Chelonia mydas*) on Ascension Island that lose on average 0.22 kg per day over the nesting season (Hays et al., 2002). This weight loss stems from the allocation of capital breeding reserves towards egg production, with no replacement of spent energetic resources. This intense energy expenditure is also hypothesised to match the production of offspring to times when survival and recruitment are the highest (Ejsmond et al., 2015; Sainmont et al., 2014; Varpe et al., 2009), further explaining the evolution of capital breeding strategies.

A key finding of our study is the interplay between maternal feeding ecology, infection status and energy allocation in nesting loggerhead turtles. Infected turtles exhibited lower body fat reserves and lower skin δ^13^C values compared to uninfected turtles. This suggests an energy trade‐off between mounting an immune response against parasites and food acquisition. Such trade‐offs between immune function and resource allocation are common across the animal kingdom (Allen & Little, 2011; Sheldon & Verhulst, 1996). While we focused on nesting females, it is likely that infection also negatively impacts the breeding frequency of females that may need more time to reach their capital breeding threshold while fighting the infection (Allen & Little, 2011). Overall our result confirms a link between feeding ecology and parasite infection in the Cabo Verde loggerhead breeding population, where turtles foraging in relatively poor oceanic environments are more likely to be infected than turtles feeding neritically or in productive oceanic upwelling areas (Lockley et al., 2020).

Because of infection and/or the likely unequal quality of feeding grounds, sea turtles could alter their energetic resource allocation to reproduction through supplementary foraging at the breeding grounds. For instance, a mixed foraging strategy has been recorded for lactating Weddle seals (*Leptonychotes weddellii*), also thought to be capital breeders, that take advantage of occasionally available prey during the reproductive season (Wheatley et al., 2008). Similarly, green turtles migrating between their Northern Cyprus breeding grounds and North African foraging grounds appear to use rich coastal waters en route to replenish resources (Godley et al., 2002). While we did not explicitly identify supplemental feeding in this study, we detected that skin and plasma δ^13^C values became more similar later in the season, which could be indicative of local feeding. Noteworthy, we observed event of feeding by nesting turtles in this population using on‐turtle cameras (BBC, Animal with Cameras 2, *personal communication*). This supplemental feeding would help top up energy stores lost to energy‐intensive breeding and nesting activities. Thus, it could be possible that, like some phocid species and even like calanoid copepods (*Calanus* spp.) (Bowen et al., 2001; Ejsmond et al., 2018), loggerhead sea turtles use a mixed strategy that starts with the use of endogenous reserves as capital breeders and then switch to an income breeding strategy later in the season, taking advantage of local resources.

Our study also revealed significant relationships between feeding ecology and reproductive output. Females with higher skin δ^15^N values, indicating either a higher trophic level or baseline‐enriched prey items, produced larger clutches (Hobson, 1999; Post, 2002; Reich et al., 2007; Zbinden et al., 2011). Alternatively, as the result of the catabolism of endogenous reserves into resources, this could represent the signal of starvation (Habran et al., 2010; Hobson et al., 1993; Voigt & Matt, 2004). Yet, this is unlikely since those high signature values are observed very early in the nesting season. Furthermore, turtles showing high skin δ^13^C values also produced bigger clutches. This positive correlation between clutch size and high δ^15^N and δ^13^C is explained by turtles foraging at more energy‐rich areas, such as oceanic areas exposed to up‐welling events (Cameron et al., 2019), which can provide more resources to allocate to reproduction (Lemos et al., 2020). Similar patterns exist in loggerhead sea turtles nesting at Zakynthos, Greece, with those foraging in the northern Mediterranean (Adriatic and Amvrakikos) showing higher δ^15^N ratios and producing larger clutches than those foraging in the southern Mediterranean (Gulf of Gabes, Coll et al., 2012; Zbinden et al., 2011). Those patterns are also seen in marine mammals such as grey seals (*Halichoerus grypus*, Sparling et al., 2006).

The benefit of an efficient foraging strategy extends beyond the direct condition of nesting turtles. Specifically, we found that the hatchlings of female loggerheads with high values of δ^13^C were heavier than those from more δ^13^C depleted mothers. Experimental tests previously showed that hatchlings with longer SCL have better swimming and dispersal abilities (Maulany et al., 2012; Scott et al., 2014). As hatchling mass correlates with length, we can postulate that hatchlings from mothers with high δ^13^C values have better dispersal capabilities (Booth & Evans, 2011; Scott et al., 2014). Infected females with high δ^13^C values produced faster offspring in both the crawl and self‐righting trials. A similar result of faster self‐righting ability correlating with maternal infection was seen by Lockley et al. (2020) for this same population. Hatchlings from females with higher δ^13^C have better dispersal abilities; this clearly demonstrates that some foraging habitats are more beneficial to individual and ultimately population health than others (Scott et al., 2014; Sorci et al., 1994).

δ^13^C and δ^15^N values, derived from skin samples proved to be a powerful tool linking feeding ecology and reproduction of sea turtles, unlike stable isotope ratios obtained from plasma samples. This discrepancy suggests that plasma samples may serve as a short‐term indicator of turtle health, reflecting more recent dietary intake, or lack thereof, while the determination of clutch size and physiological condition occurs before the nesting season commences. These findings align with previous studies highlighting the differential information provided by skin and plasma samples in stable isotope analysis (Hobson, 1999; Reich et al., 2007; Zbinden et al., 2011).

Overall, our study showed that individual female turtles that likely forage in more productive foraging grounds have increased reproductive output and produce hatchlings of higher quality. We further uphold the suggestion that turtles may be able to switch from a capital breeding strategy to an income strategy using supplementary local feeding.

## AUTHOR CONTRIBUTIONS

**Leila Fouda:** Conceptualization (equal); data curation (lead); formal analysis (lead); funding acquisition (equal); investigation (lead); methodology (equal); project administration (equal); software (lead); validation (lead); visualization (lead); writing – original draft (lead); writing – review and editing (lead). **Stuart R. B. Negus:** Data curation (supporting); investigation (supporting); writing – review and editing (supporting). **Emma C. Lockley:** Data curation (supporting); funding acquisition (supporting); investigation (supporting); writing – review and editing (supporting). **Kirsten Fairweather:** Investigation (supporting); resources (supporting); writing – review and editing (supporting). **Artur Lopes:** Investigation (supporting); resources (supporting); writing – review and editing (supporting). **Anice Lopes:** Investigation (supporting); writing – review and editing (supporting). **Sandra M. Correia:** Project administration (supporting); writing – review and editing (supporting). **Albert Taxonera:** Data curation (supporting); investigation (supporting); project administration (supporting); resources (supporting); writing – review and editing (supporting). **Gail Schofield:** Supervision (supporting); writing – review and editing (supporting). **Christophe Eizaguirre:** Conceptualization (equal); data curation (supporting); formal analysis (supporting); funding acquisition (equal); investigation (supporting); methodology (equal); project administration (equal); resources (lead); supervision (lead); writing – review and editing (equal).

## CONFLICT OF INTEREST STATEMENT

The authors declare no competing interests.

## STATEMENT ON INCLUSION

Our study brings together authors from many different countries and includes contributions from authors in Cabo Verde where our study was conducted. Authors were engaged in data collection and study design and our in‐country collaborators made the large‐scale fieldwork and sample collection possible. Our research is in collaboration with local NGOs and communities and where relevant the literature published by scientist in the region was cited.

## Supporting information


Appendix S1.

## Data Availability

All data and code for this study are available on Dryad under Doi: 10.5061/dryad.vx0k6dk07.
