# Supplementary material for: Productive foraging grounds enhance maternal condition and offspring quality in a capital breeding species
Source: Ecol Evol. 2024 Sep 10;14(9):e70137. doi: 10.1002/ece3.70137 (PMC11387723; doi:10.1002/ece3.70137)
Supplement: Supplementary file 1 — Appendix S1. [file ECE3-14-e70137-s001.pdf]

Supplementary Material for:

Productive foraging grounds enhance maternal condition and offspring quality in a capital breeding species

This file includes:

Supplementary Figure S1

Supplementary Tables S1 to S3

Figure S1: A biplot of stable isotope values ($\delta^{13}C$ and $\delta^{15}N$) in the two tissue types (skin and blood plasma) over the 2018 nesting season on Sal.

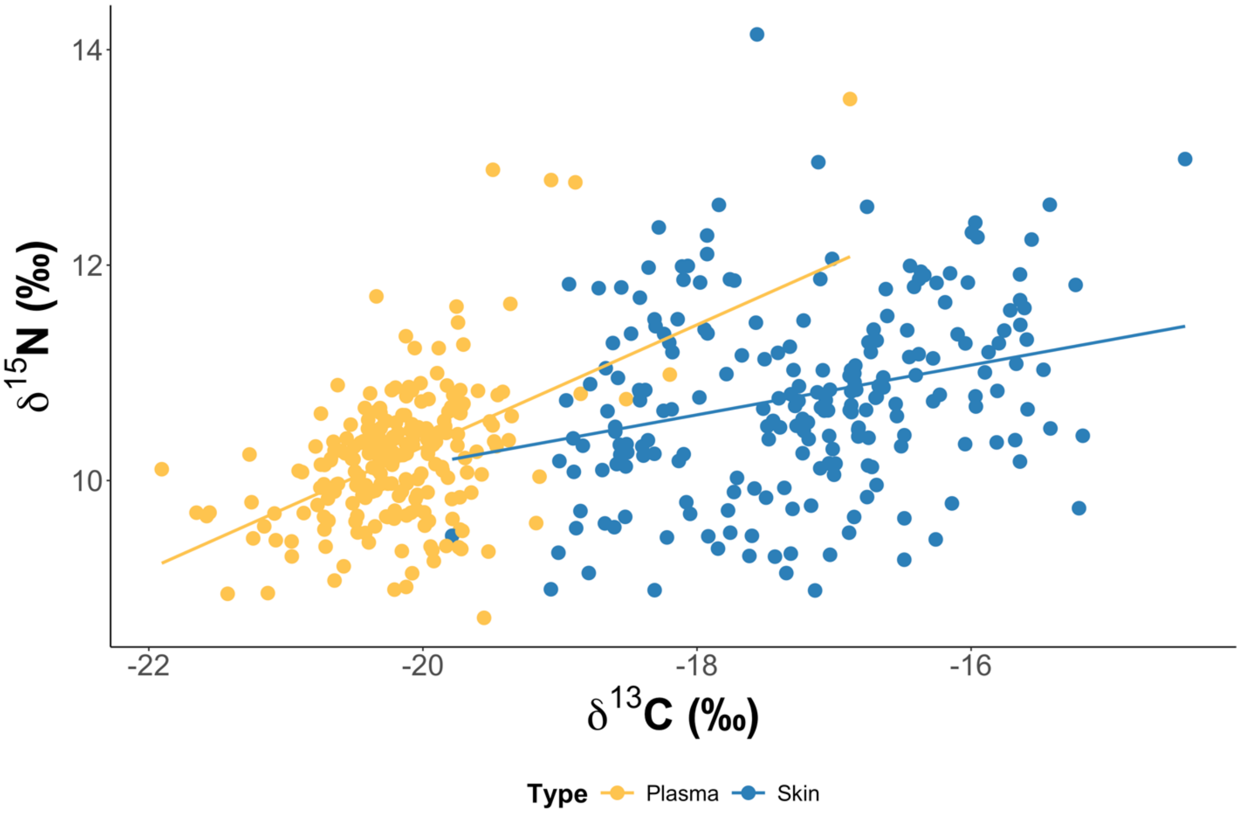

Table S1: Summary for linear model results for changes to stable isotope values ($\delta^{13}C$ and $\delta^{15}N$) in the two tissue types (skin and blood plasma) over the 2018 nesting season on Sal. Significant values in bold.

Variable	d.f.	F	p
**$\delta^{13}C$**			
Skin~Julian Date	1, 237	0.093	0.76
Plasma~Julian Date	1, 210	22.206	**<0.001**
$\delta^{13}C$~Type	1, 449	1424.8	**<0.001**
**$\delta^{15}N$**			
Skin~Julian Date	1, 237	24.675	**<0.001**
Plasma~Julian Date	1, 210	10.315	**0.002**
$\delta^{15}N$~Type	1, 449	66.579	**<0.001**

Table S2: Summary table for best reduced models investigating the correlations of nesting turtle characteristics on stable isotope determinants of $\delta^{13}C$ and $\delta^{15}N$ in the skin and blood samples. All models were backwards-selected using AIC. *d.f* denotes degrees of freedom. Significant values in bold.

Variables	*d.f.*	F	*p*
**Skin $\delta^{13}C$**			
Fat Reserves	1,207	0.417	0.519
Parasite Presence	1,207	0.139	0.709
CCL	1,207	6.665	**0.011**
Nesting Season Period	2,207	3.215	**0.042**
Fat Reserves:Parasite Presence	1,207	5.013	**0.026**
Fat Reserves:Nesting Season Period	2,207	7.695	**0.001**
**Skin $\delta^{15}N$**			
Fat Reserves	1,208	0.484	0.488
Parasite Presence	1,208	0.073	0.787
CCL	1,208	0.17	0.681
Nesting Season Period	2,208	12.265	**<0.001**
Fat Reserves:CCL	1,208	5.717	**0.018**
Parasite Presence:CCL	1,208	4.054	**0.045**
**Plasma $\delta^{13}C$**			
Fat Reserves	1,197	1.031	0.311
Parasite Presence	1,197	0.012	0.915
CCL	1,197	0.222	0.638
Nesting Season Period	2,197	17.715	**<0.001**
**Plasma $\delta^{15}N$**			
Fat Reserves	1,196	3.528	0.062
Parasite Presence	1,196	3.635	0.058
CCL	1,196	2.165	0.143
Nesting Season Period	2,196	6.538	**0.002**
Fat Reserves:CCL	1,196	16.125	**<0.001**

Table S3: Summary table reporting the best-reduced model testing the effects of maternal feeding ecology via skin stable isotopes and fitness on hatchling health. All models were backwards-selected using AIC. *d.f* denotes degrees of freedom. Significant values in bold.

Variables	*d.f.*	F	p
**Hatchling Length**			
Fat Reserves	1,73	7.287	**0.009**
Parasite Presence	1,73	0.083	0.774
Skin $\delta^{13}C$	1,73	3.611	0.061
Skin $\delta^{15}N$	1,73	1.438	0.234
CCL	1,73	12.912	**0.001**
Clutch Size	1,73	9.972	**0.002**
Incubation Duration	1,73	1.252	0.267
**Hatchling Mass**			
Fat Reserves	1,70	12.739	**0.001**
Parasite Presence	1,70	0.056	0.813
Skin $\delta^{13}C$	1,70	7.149	**0.009**
Skin $\delta^{15}N$	1,70	0.120	0.73
CCL	1,71	7.702	**0.007**
Clutch Size	1,71	1.114	0.295
SCL	1,1595	681.202	**<0.001**
Incubation Duration	1,70	2.316	0.133
Fat Reserves:SCL	1,1586	42.526	**<0.001**
CCL:Clutch Size	1,70	5.413	**0.023**
**Hatchling Crawl Speed**			
Fat Reserves	1,70	7.297	**0.009**
Parasite Presence	1,69	7.179	**0.009**
Skin $\delta^{13}C$	1,69	5.438	**0.023**
Skin $\delta^{15}N$	1,69	0.0001	0.994
CCL	1,70	0.947	0.334
Clutch	1,69	3.329	0.072
Hatchling Body Mass Index	1,1267	5.655	**0.018**

Incubation Duration	1,69	5.405	**0.023**
Fat Reserves: Skin $\delta^{15}N$	1,69	11.767	**0.001**
Parasite Presence: Skin $\delta^{13}C$	1,69	7.814	**0.007**
Skin $\delta^{13}C$:Incubation Duration	1,69	5.734	**0.019**
Clutch Size:Incubation Duration	1,69	3.915	0.052
**Hatchling Self-Righting Speed**			
Fat Reserves	1,70	0.121	0.729
Parasite Presence	1,69	6.15	**0.016**
Skin $\delta^{13}C$	1,70	0.257	0.613
Skin $\delta^{15}N$	1,69	0.145	0.705
CCL	1,72	0.093	0.761
Clutch	1,69	0.006	0.937
Hatchling Body Mass Index	1,1190	6.003	**0.014**
Incubation Duration	1,69	6.532	**0.013**
Parasite Presence: Skin $\delta^{13}C$	1,69	5.622	**0.021**
Parasite Presence:Clutch Size	1,69	6.747	**0.011**

Table S4: Summary table reporting the best-reduced model testing the effects of maternal feeding ecology via plasma stable isotopes and fitness on hatchling health. All models were backwards-selected using AIC. *d.f* denotes degrees of freedom. Significant values in bold.

Variables	*d.f.*	F	p
**Hatchling Length**			
Fat Reserves	1,68	3.403	0.069
Parasite Presence	1,68	0.054	0.816
Plasma δ13C	1,68	6.979	**0.010**
Plasma δ15N	1,68	0.417	0.521
CCL	1,68	13.678	**<0.001**
Clutch Size	1,68	9.556	**0.003**
Incubation Duration	1,68	1.826	0.181
Plasma δ13C:Plasma δ15N	1,68	0.444	0.508
**Hatchling Mass**			
Fat Reserves	1,67	4.506	**0.037**
Parasite Presence	1,68	5.513	**0.022**
Plasma δ13C	1,68	5.492	**0.022**
Plasma δ15N	1,67	3.224	0.077
CCL	1,70	18.142	**<0.001**
Clutch Size	1,67	0.854	0.359
SCL	1, 1524	16.363	**<0.001**
Incubation Duration	1,67	2.739	0.103
Fat Reserves:SCL	1, 1505	13.798	**<0.001**
Parasite Presence:CCL	1,68	5.567	**0.021**
Plasma δ13C:SCL	1, 1524	11.804	**0.001**
Plasma δ15N:SCL	1, 1523	14.191	**<0.001**
**Hatchling Crawl Speed**			
Fat Reserves	1,65	8.726	**0.004**
Parasite Presence	1,65	3.483	0.067
Plasma δ13C	1,66	3.655	0.060
Plasma δ15N	1,65	0.184	0.669

CCL	1,67	2.758	0.101
Clutch Size	1,65	12.278	**<0.001**
Hatchling Body Mass Index	1, 1163	4.134	**0.042**
Incubation Duration	1,65	1.347	0.250
Fat Reserves:Incubation Duration	1,65	8.643	**0.005**
Parasite Presence:Incubation Duration	1,65	3.620	0.062
Clutch Size:Incubation Duration	1,65	13.441	**<0.001**
**Hatchling Self-Righting Speed**			
Fat Reserves	1,66	1.013	0.318
Parasite Presence	1,66	1.775	0.187
Plasma δ13C	1,68	6.424	**0.014**
Plasma δ15N	1,68	7.224	**0.009**
CCL	1,70	0.110	0.741
Clutch Size	1,66	4.017	**0.049**
Hatchling Body Mass Index	1, 1071	5.570	**0.018**
Incubation Duration	1,67	1.780	0.187
Clutch Size:Incubation Duration	1,66	4.466	**0.038**
